# Genomic Characterization of Chordoma: Insights from the AACR Project GENIE Database

**DOI:** 10.3390/cancers17030536

**Published:** 2025-02-05

**Authors:** Beau Hsia, Gabriel Bitar, Saif A. Alshaka, Jeeho D. Kim, Bastien A. Valencia-Sanchez, Farhoud Faraji, Michael G. Brandel, Mariko Sato, John Ross Crawford, Michael L. Levy, Vijay A. Patel, Sean P. Polster

**Affiliations:** 1 School of Medicine, Creighton University, Phoenix, AZ 85012, USA; beauhsia@creighton.edu (B.H.);; 2 Naval Medical Center San Diego, Department of Otolaryngology-Head and Neck Surgery, San Diego, CA 92134, USA; 3 Department of Otolaryngology—Head and Neck Surgery, Mayo Clinic Florida, Jacksonville, FL 32224, USA; 4 Division of Pediatric Otolaryngology, Rady Children’s Hospital, San Diego, CA 92123, USA; 5 Department of Neurosurgery, University of California San Diego—Rady Children’s Hospital, San Diego, CA 92123, USA; 6 Department of Pediatric Oncology, Children’s Hospital of Orange County, Orange, CA 92868, USA; 7 Department of Pediatrics and Neurology, Children’s Hospital Orange County, University of California Irvine, Orange, CA 92868, USA; 8 Department of Otolaryngology—Head and Neck Surgery, University of California San Diego, San Diego, CA 92093, USA; 9 Department of Neurosurgery, University of Chicago, Chicago, IL 60637, USA

**Keywords:** AACR project GENIE, chordoma, genomic alterations, SWI/SNF complex mutations, chromatin modification, biomarker discovery, targeted therapy, cancer genomics

## Abstract

**Background:** Chordoma is a rare primary tumor originating from embryonic notochord remnants, with limited systemic therapeutic options due to a poor understanding of its genomic landscape. This study aims to characterize the genetic alterations in chordoma using a large national patient-level genomic repository, the AACR Project GENIE, to identify potential therapeutic targets and improve disease modeling. **Methods:** A retrospective analysis of chordoma samples was conducted using the AACR Project GENIE database. Targeted sequencing data were analyzed for recurrent somatic mutations, tumor mutational burden, and chromosomal copy number variations, with significance set at *p* < 0.05. **Results:** Frequent mutations were observed in genes associated with SWI/SNF complex affecting chromatin remodeling (*SETD2*, *PBRM1*, *ARID1A*). Mutations were also common among the *TERT* promoter regions, and cell cycle regulation (*CDKN2A*). Significant co-occurrences were identified among *PBRM1*, *BRCA2*, and *KMT2D* mutations. *CDKN2A/B* deletions were enriched in metastatic tumors, and pediatric cases demonstrated distinct mutation profiles compared to adults. **Conclusions:** This study provides a genomic profile of chordoma, identifying key mutations and potential therapeutic targets. These findings highlight the roles of chromatin remodeling and cell cycle pathways in chordoma biology, offering insights for future precision medicine approaches and therapeutic interventions.

## Simple Summary

Chordoma is a rare, slow-growing tumor arising from remnants of the embryonic notochord. This study investigates genomic alterations in chordoma using a large national patient-level repository, the AACR Project GENIE. The goal was to identify mutations in key genes such as *PBRM1*, *ARID1A*, *TERT*, and *TP53*, which may influence tumor behavior, treatment response, and clinical outcomes. These findings contribute to an improved understanding of chordoma biology, highlighting potential biomarkers and therapeutic targets.

## 1. Introduction

Chordoma is a slow-growing, indolent neoplasm arising from embryonic notochord remnants along the neuraxial skeleton, most commonly in the sacrum and skull base. Chordoma is rare, with an age-adjusted incidence rate (IR) of 0.08 per 100,000 individuals [1]. It is more common in males (IR 0.10) and in White and Asian/Pacific Islander patients [2,3], with a peak incidence in the 75–84 year age group, and less than 5% of cases are pediatric [3,4]. The reported median survival for chordoma patients is 6.3 years, with 5- and 10-year survival rates at 67.6% and 39.9%, respectively [2]. Chordoma presents significant surgical challenges due to its proximity to critical neurovascular structures. Patients with chordoma often present with a variety of symptoms contingent on its anatomical location, from local invasion or mass effect, while some are discovered incidentally [5,6].

Surgical resection, often with adjuvant radiation early in the disease course, is the standard first-line treatment for chordoma. The current treatment paradigm for chordoma prioritizes maximal resection while minimizing morbidity. Factors such as tumor location, adjacent neurovascular structures, functional status, and patient preferences guide the extent of the resection [7]. Gross total resection (GTR) is generally considered one of the most important prognostic factors for prolonging overall survival and reducing the risk of locoregional recurrence [7]. Recurrence often portends a poor prognosis and is managed with re-resection, if possible, or with high-dose conformal radiation therapy, as chordoma is known for its unresponsiveness to traditional chemotherapeutics [8,9]. This is in contrast to other solid tumors, which are increasingly managed with systemic therapy in the salvage setting as the disease progresses.

At present, there are no FDA-approved systemic therapeutic agents for patients diagnosed with chordoma [10,11], likely due to insufficient understanding of its molecular pathobiology. Therefore, furthering our understanding of the tumor biology in chordoma may facilitate the initiation of novel clinical trials or corroborate the use of current experimental therapies, potentially introducing systemic therapies into the therapeutic paradigm for chordoma management. A recent review by Chen et al. has highlighted ongoing clinical trials exploring various systemic therapies, including targeted therapy, immunotherapy, and chemotherapy, which may offer new treatment avenues for chordoma patients [6].

The current biological understanding of chordoma is based on the tumor’s origin from notochordal remnants, with conventional, chondroid, and dedifferentiated subtypes, among others. Studies show that for chordoma, 8% and 1% are represented by the chondroid and dedifferentiated subtypes, respectively [3].

Recent studies have identified recurrent mutations in the brachyury gene (TBXT) in chordoma, with up to 70% of tumors showing duplications of this gene. Additionally, mutations in genes involved in the PI3K/AKT/mTOR pathways have been found in a subset of chordoma, particularly those arising from the skull base. Some chordomas also exhibit alterations in genes related to chromatin remodeling, such as *ARID1A* and *PBRM1*. Alterations in chromatin remodeling genes can disrupt the regulation of gene expression by modifying the accessibility of transcriptional machinery to DNA, potentially driving oncogenic processes in chordoma and influencing its biological behavior and therapeutic response [12]. Additional studies have found that 1p36 and 9p21 deletions affect phenotypic mitotic activity, as well as response to radiation therapy [13].

However, despite these advances, the full spectrum of genomic alterations in chordoma is not yet fully characterized, and secondary drivers that may contribute to tumor progression or treatment resistance remain an active area of research. Thus, through the utilization of a publicly accessible repository of patient-level data, this study aims to characterize the somatic mutational landscape of chordoma to inform new therapeutic approaches and improve disease modeling.

## 2. Materials and Methods

This study was deemed exempt from institutional review board approval at Creighton University, as the database is deidentified and publicly available. The American Association for Cancer Research (AACR) Project Genomics Evidence Neoplasia Information Exchange (GENIE)^®^ database was accessed using the cBioPortal (v17.0-public) online software [14] on 21 January 2025, with clinical data dating back to 2017. Genomic sequencing information from 19 international cancer centers is compiled in the AACR GENIE^®^ repository. The dataset comprises heterogeneous sequencing platforms, including whole-genome sequencing (WGS), whole-exome sequencing (WES), and targeted gene panels (50–555 genes). Approximately 80% of samples were sequenced using targeted panels, 15% via WES, and 5% via WGS. Sequencing depth varied by platform: targeted panels achieved >500× coverage, WES ~ 150×, and WGS ~ 30×. Of the total samples, 65% were tumor-only sequencing, while 35% included matched normal tissues for germline variant filtering.

Participating institutions use institution-specific pipelines for mutation calling and annotation, though all adhere to GENIE’s harmonization protocols (e.g., GATK for variant detection, and ANNOVAR for annotation). Only a select number of cancer types include therapeutic response along with clinical outcomes data, but treatment regimens were not recorded for chordoma. Additionally, each participating institution may use different pipelines from each other (and within the same institution). Participating institutions use either unbiased whole genomic/exome sequencing or targeted panels of up to 555 genes. 

All patients with bone tumors were queried for a pathologic diagnosis of chordoma. Primary tumor samples are derived from the original site of tumorigenesis, whereas metastatic samples are obtained from sites of distant disease dissemination. Differences in mutation frequencies between primary and metastatic tumors were investigated by calculating the proportion of samples harboring mutations in each gene for both groups and comparing them using a chi-squared test. The dataset included genomic data (e.g., somatic mutations), histological subtype, as well as clinical demographics (e.g., race, sex, and age). Histologic subtypes were classified per WHO criteria, including ‘Chordoma NOS’ for cases lacking definitive morphologic or molecular features for specific subtypes, as subtyping is often precluded by insufficient tissue or ambiguous pathology in the clinical setting. Targeted panel compositions varied across institutions, with common cancer-associated genes (e.g., PIK3CA, EGFR, KRAS) included in the majority of panels. However, non-druggable or rare cancer genes were absent from the panels. Structural variants were excluded from this analysis. Copy number alterations (CNAs), including homozygous deletions and amplifications, were assessed and frequencies of recurrent CNAs were calculated. Tumor mutational burden was calculated based on the number of detected somatic mutations (synonymous and nonsynonymous) per megabase (Mb) of sequenced DNA. For panel-based TMB, values were normalized to panel size (e.g., a 1.5 Mb panel’s TMB = [total mutations/1.5]) and adjusted using GENIE’s regression models to approximate WES-equivalent TMB. This approach accounts for differences in panel size and variant allele frequency cutoffs (≥5% threshold). Samples with missing data were excluded. To assess the novelty of recurrent mutations, variants were cross-referenced with the Catalogue of Somatic Mutations in Cancer (COSMIC v101, accessed on 29 January 2025) using gene-specific queries. Statistical analyses were conducted using R/R Studio (R Foundation for Statistical Computing, Boston, MA, USA), with significance set at *p*  <  0.05. Continuous variables were reported as means ± standard deviations (SD), and categorical variables were presented as frequencies and percentages. Differences between categorical variables were assessed using the chi-squared test. For comparisons of means between two groups, a two-sided *t*-test and nonparametric tests, such as the Mann–Whitney U test for non-normally distributed data, were applied. The Benjamini–Hochberg False Discovery Rate (FDR) correction was used to adjust for multiple comparisons.

Somatic mutations were filtered to include nonsynonymous variants (missense, nonsense, frameshift, and splice site) with a variant allele frequency (VAF) ≥ 5% and sequencing coverage ≥ 100×. Synonymous mutations and variants of unknown significance (VUS) were excluded. Mutation calls were derived from GENIE’s harmonized Mutation Annotation Format (MAF) files, which standardize variant annotation (e.g., gene symbol, protein change) across contributing institutions.

## 3. Results

### 3.1. Chordoma Patient Demographics

Given the rarity of chordoma and the limited sample size in genomic cohorts, we prioritized general demographic analysis of chordoma, grouping together primary and metastatic tumors. Eighty-five (72.0%) patients were White, eight (6.8%) were Asian, and seven (5.9%) were Black. Ninety-six (81.4%) patients identified as Non-Hispanic, eleven (9.3%) had unknown ethnicity, and ten (8.5%) were of Hispanic descent. Sixty-six (55.4%) patients were male and fifty-three (44.9%) were female. There were no significant demographic or genomic differences between pediatric and adult patients.

Chordoma patient demographics are described in detail in Table 1. Of 1437 bone cancer samples collected, 133 (9.2%) were chordoma, including 14 (10.5%) conventional, 3 (2.3%) dedifferentiated, and 116 (87.2%) unspecified subtypes. Eighteen (15.2%) patients were pediatric (age < 18 years) and the rest were adults (*n* = 115; 84.8%).

Among adult patients, the average age was 55.7  ±  17.1 years. Moreover, 67 (50.4%) samples sequenced were of the primary tumor and 54 (40.6%) were from metastases. The tumor mutational burden across the entire cohort was 4.2  ±  3.1 mutations per megabase (mut/Mb).

### 3.2. Somatic Mutations and Copy Number Alterations

**Figure 1:**
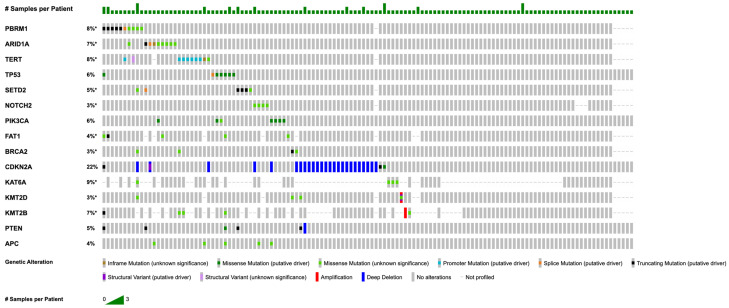
OncoPrint of recurrent mutations in chordoma (for genes with n ≥ 5, VAF ≥ 5%, coverage ≥ 100×). Star (*) indicates that not all samples were profiled.

The most frequently observed mutations are detailed in Figure 1. Notably, each of these genes (e.g., *PBRM1*, *SETD2*, *NOTCH2*) included at most one instance in which two samples from the same patient contributed to its mutation count. The exclusion of these duplicate entries did not alter the list of the top 15 genes. The majority (77.8%) of *TERT* mutations were the 5′flank type, resulting in promotor changes, and *ARID1A* (70.0%), *TP53* (87.5%), and *NOTCH2* (100%) were associated with missense mutations. *PBRM1* mutations tended to co-occur with *BRCA2* (2/10; *p* = 0.027) and *KMT2D* (2/10; *p* = 0.044), while *KMT2D* mutations tended to co-occur with *BRCA2* (*n* = 3/6; *p* < 0.001). There were no additional statistically significant mutual co-occurrences or mutual exclusivities. *ARID1A*, *TERT*, *TP53*, *SETD2*, and *NOTCH2* were relatively mutually exclusive with only one (<0.1%) sample with more than one of these mutations. 

In addition to somatic mutations, we identified recurrent copy number alterations (CNAs) in 99 samples. The loss of heterozygosity (LOH) events was prevalent, particularly affecting tumor suppressor genes, such as *CDKN2A* (*n* = 27; 27.3%)*, CDKN2B* (*n* = 25; 25.3%), and *SMARCB1* (*n* = 7; 7.1%). Amplifications were less frequent, observed in genes such as *CBFB* (*n* = 4; 4.2%), *CDH1* (*n* = 3; 3.0%), *ETV1* (*n* = 2; 2.0%), and *CTCF* (*n* = 2; 2.1%).

### 3.3. Chordoma SWI/SNF Complex Mutations

The following analysis investigated different point mutations, or specific amino acid substitutions or deletions, in the most common SWI/SNF complex mutations (*PBRM1*, *ARID1A*, or *SETD2*) listed in Table 2. 

Across the three genes, a variety of mutation types appear, with a notable frequency of frameshift insertions (FS ins) and missense mutations. *PBRM1* shows a mix of FS insertions (*n* = 2), nonsense (*n* = 2), and missense mutations (*n* = 4), while *ARID1A* has a high frequency of missense mutations (*n* = 7)*. SETD2* exhibits a combination of nonsense (*n* = 2), missense (*n* = 2), FS insertion (*n* = 2), and splice site mutations (*n* = 1), possibly suggesting different mechanisms of inactivation across the genes in the SWI/SNF complex.

In *SETD2*, the T2338Hfs*31 mutation appears recurrently in exon regions (*n* = 2), which may indicate mutation hotspots within specific functional domains of the protein, potentially impacting chromatin modification functions critical to cancer biology in chordoma. In *PBRM1*, the recurrence of the missense mutation D1055Y (*n* = 2) and the nonsense mutation W1417* (*n* = 2) in samples suggest these particular variants may be biologically significant in chordoma.

Notably, recurrent mutations in *SETD2* (e.g., T2338Hfs31 in exon regions, *n* = 2) and *PBRM1* (e.g., D1055Y, *n* = 2; W1417, *n* = 2) were absent from the COSMIC database’s chordoma entries, indicating potential novel hotspots.

### 3.4. Chordoma NOS, Conventional Type Chordoma, and Dedifferentiated Chordoma Subtypes Mutational Landscape

The patient demographics stratified by histology subtype are illustrated in Table 3. In this cohort, composed of 116 patients with chordoma NOS, 14 with conventional chordoma, and 3 with dedifferentiated chordoma, mutations in SWI/SNF complex genes (*PBRM1*, *ARID1A*, *SETD2*) were not significantly enriched or depleted across subtypes (*p* > 0.05). *PBRM1* mutations were present in 8.3% (*n* =  9/116) of chordoma NOS cases and 7.1% (*n* =  1/14) of conventional chordoma, while *ARID1A* mutations appeared in 6.4% (*n* =  7/116) of chordoma NOS cases and 14.3% (*n* =  2/14) of conventional chordoma. *SETD2* mutations were observed in 6.4% (*n* =  7/116) of chordoma NOS cases but were absent in other subtypes. Significant enrichments were identified in dedifferentiated chordoma, specifically for *CARM1* (100%, *p* < 10^−10^), *PRKN* (66.67%, *p* < 10^−10^), and *MTAP* (100%, *p* = 0.009), suggesting these mutations may uniquely characterize dedifferentiated chordoma.

### 3.5. Adult vs. Pediatric Mutational Landscape

The most common mutations in pediatric chordoma cases were *MRE11*, *FANCA*, and *MSH6*, each individually appearing in two of the pediatric samples. Most pediatric cases (*n* = 8) were identified as chordoma NOS. In contrast, adult chordoma cases, primarily within the 121-sample cohort, showed a higher frequency of mutations in *PBRM1* (*n* = 10), *ARID1A* (*n* = 9), *TP53* (*n* = 8), *TERT* (*n* = 8), and *SETD2* (*n* = 7). Pediatric cases had no SWI/SNF complex mutations, a notable difference from adults, where such mutations were more prevalent. Specific mutations, such as *MRE11* (*p* < 0.01) and *ARID5B* (*p* < 0.05), were significantly enriched in pediatric chordoma compared to adults, highlighting potential age-specific genetic differences in chordoma. The demographic differences between adult and pediatric chordoma are highlighted in Table 4.

### 3.6. Primary vs. Metastatic Disease Mutational Landscape

There were 67 primary chordoma cases, and mutations were observed in *ARID1A* (*n* = 5; 7.5%), *PIK3CA* (*n* = 5; 7.5%), *TP53* (*n* = 4; 6.0%), and *PBRM1* (*n* = 4; 6.0%), as seen in Table 5. In contrast, 54 metastatic chordoma displayed mutations in *SETD2* (*n* = 6; 11.1%), *BRCA2* (*n* = 5; 9.3%), *PBRM1* (*n* = 5; 9.3%), and *ARID1A* (*n* = 4; 7.4%). The cohort sizes of primary (*n* = 67) and metastatic (*n* = 54) tumors were comparable, reducing potential bias from unbalanced group sizes. Notably, *BRCA2* mutations were significantly enriched in metastatic tumors (9.3% vs. 0%; *p* = 0.0138), as were *SETD2* mutations (11.1% vs. 1.5%; *p* = 0.0411). However, the overall mutational landscape—including tumor mutational burden (TMB) and recurrent alterations in key genes like ARID1A and PBRM1—was largely overlapping between groups.

## 4. Discussion

This work aimed to profile the somatic mutational landscape of chordoma using a publicly available genomic repository. Overall, as it has been previously demonstrated [15], the AACR Project GENIE^®^ repository is distinct from TCGA and provides a new platform for biomarker discovery in rare tumor subtypes, where novel targets may be identified for future precision therapy. 

It appears that a subset (20.3%) of chordoma is enriched with at least one SWI/SNF complex mutation: *PBRM1*, *ARID1A*, or *SETD2*. Additionally, similar to other reports, mutations in *PBRM1* are found mostly in the chordoma NOS or conventional subtype [16]. Interestingly, chordoma mutations in the *PBRM1*, *ARID1A*, or *SETD2* genes are frequently missense mutations. Furthermore, *PBRM1* mutations significantly co-occur with *BRCA2* and *KMT2D*, genes associated with DNA repair mechanisms and regulation of gene expression by modifying chromatin structure, impacting cell growth, differentiation, and survival [17,18]. The co-occurrence of *PBRM1* with these secondary drivers may indicate a collaborative disruption of DNA repair and chromatin remodeling pathways, which are essential for maintaining cellular integrity. The recurrence of specific mutations could indicate a role in tumorigenesis or progression, underscoring these as potential targets for further research.

Previous studies have described the somatic mutational landscape of chordoma, particularly for the aforementioned histologic subtypes. Specifically, with immunohistochemistry, chordoma can be diagnosed with positive staining for brachyury, a key transcription factor encoded by the *TBXT gene* [19]. 

Pediatric and adult chordoma exhibit distinct genetic profiles, which align with their dissimilar prognoses, with a pediatric survival rate ranging from 56.8 to 81%, while adults survive at a rate of 23 to 66% [20]. While there are genetic alterations shared between age groups, recent studies have identified several important differences. Pediatric chordoma is characterized by a higher prevalence of germline *ARID1B* indels (22% vs. 5% in adults) and more frequent loss of *INI1* (*SMARCB1*) [21,22], especially in poorly differentiated cases. In contrast, adult chordoma more commonly displays *PBRM1* alterations and homozygous deletions of the *CDKN2A*/*2B* locus [23], reflecting the findings in this study. This underscores the importance of age-specific approaches in both research and clinical management of chordoma, while also recognizing shared features that could inform broader therapeutic strategies.

Profiling the mutational landscape in chordoma has therapeutic implications. Despite a relatively low mutational burden, chordomas harbor specific alterations that can be targeted therapeutically [23]. For instance, mutations in PI3K pathway genes, particularly *PIK3CA*, have been associated with shorter progression-free survival and may be targetable with PI3K inhibitors [16]. Additionally, homozygous deletions of *CDKN2A* and *CDKN2B* genes, which are common in chordoma, suggest potential susceptibility to CDK4/6 inhibitors [24]. 

Our findings of enrichments in SWI/SNF complex mutations, such as *PBRM1*, *ARID1A*, *SETD2*, and *SMARCA2*, indicate that epigenetic therapies may be effective in some chordoma cases [23]. The identification of these genetic alterations provides further evidence for molecular testing of chordoma patients to guide treatment selection and develop personalized therapeutic strategies.

SWI/SNF complex mutations are prevalent across a wide range of human cancers, occurring in approximately 20% of all malignancies [25,26]. These mutations affect various subunits of the SWI/SNF chromatin remodeling complex, with ARID1A being the most frequently mutated gene, followed by *SMARCA4*, *ARID1B*, *ARID2*, *PBRM1*, and *SMARCB1* [27,28], mutations we have found to be common in adult chordoma. The spectrum of SWI/SNF mutations varies among different cancer types. Endometrial cancer, gallbladder and biliary tract cancer, and gastric cancer exhibit particularly high rates of SWI/SNF mutations, with frequencies of 54.1%, 43.4%, and 33.9%, respectively [27]. Other cancer types with notable SWI/SNF mutation rates include urothelial cancer, ovarian and fallopian tube cancer, and non-small cell lung cancer [27,28].

Importantly, SWI/SNF mutations have been associated with specific cancer vulnerabilities and therapeutic opportunities. Tumors with SWI/SNF mutations often exhibit higher tumor mutational burden (TMB) and may respond better to immune checkpoint inhibitor (ICI) treatment [27,29]. This association has led to increased interest in exploring immunotherapy approaches for patients with SWI/SNF-mutant cancers [28,29].

SWI/SNF mutations can impact cancer differentiation and response to targeted therapies. For instance, in thyroid cancer, SWI/SNF complex mutations have been shown to promote dedifferentiation and resistance to *MAPK* inhibitor-based redifferentiation therapies [30]. Despite the prevalence and potential therapeutic implications of SWI/SNF mutations, directly targeting the mutated complex remains challenging. Current research focuses on exploiting synthetic lethal interactions and exploring combination therapies that leverage the vulnerabilities created by SWI/SNF deficiency [28,29].

Since *EZH2* is often overexpressed in SWI/SNF-mutant cancers, it makes for a potential target for therapies, as is the case with *EZH2* inhibitors, currently in clinical trials [6]. As SWI/SNF plays a role in regulating gene expression and cellular processes, *HDAC* inhibitors may be able to help restore normal gene expression patterns by modifying histone acetylation in SWI/SNF-mutated tumors [31]. *BET* inhibitors—drugs that currently target bromodomain proteins involved in chromatin remodeling and gene regulation—may also be promising.

### Limitations

This study has several limitations. First, this database lacks transcriptomic information, preventing mutational status from being correlated with downstream pathway activity or gene expression levels. Second, this database does not include treatment information, which would allow analysis of treatment response with mutational status and histologic subtype. GENIE’s lack of treatment data also precludes analysis of therapy-related genomic changes that may confound comparisons between primary and metastatic tumors. Third, given that different centers use various sequencing platforms, our results may either be over- or underestimating the true top mutation frequencies in chordoma, along with the frequencies of the secondary driver mutations. Fourth, this study does not include methylation analysis, which plays a critical role in the epigenetic regulation of gene expression in chordoma. Methylation changes could potentially provide further insights into tumor biology and therapeutic resistance. Fifth, the sample size is relatively small, which limits the statistical power to detect associations between specific mutations and clinical outcomes or other disease characteristics. Future studies with larger, uniformly annotated cohorts are necessary to establish independent prognostic associations. Sixth, the inclusion of a “not otherwise specified” (NOS) category for histologic subtype poses a limitation to our analysis. This category encompasses tumors with uncharacterized molecular or morphologic features, potentially obscuring true subtype-specific differences. Future studies with larger sample sizes and more detailed histological characterization may allow for a more refined analysis of these subtypes. Seventh, the absence of longitudinal sampling (e.g., paired primary–metastatic samples from the same patient) restricts our ability to distinguish driver mutations from passenger events acquired during progression. Eighth, the GENIE database includes a small proportion of non-independent samples (e.g., primary and metastatic tumors from the same patient). However, this analysis (see Results) indicates that this has a negligible impact on our overall findings. Ninth, the absence of survival data (e.g., OS, DFS) in the GENIE database prevented the correlation of mutational status with clinical outcomes. Future studies combining genomic profiling with longitudinal survival data are warranted to evaluate the prognostic implications of recurrent mutations in chordoma. Lastly, we could not correlate mutational status with immunohistochemical expression of various tumor-intrinsic or immune-related markers. Despite these limitations, this study provides novel information on SWI/SNF complex mutations in chordoma and may lead to novel pre-clinical model development for targeted therapy testing.

## 5. Conclusions

Mutations in *PBRM1*, *ARID1A*, and *SETD2* appear to be enriched in chordoma. Given the roles of these genes in chromatin remodeling, their frequent mutation points to a broader vulnerability in the SWI/SNF complex in chordoma, which may present novel opportunities for targeting chromatin regulation pathways in future therapeutic strategies. This study is important for advancing chordoma preclinical models, diagnostic testing, and targeted therapy development.

## Figures and Tables

**Table 1:** Chordoma patient demographics.

Demographics	Category	N (%)
	Conventional Chordoma	14 (10.5)
Histology	Dedifferentiated Chordoma	3 (2.3)
	Not Otherwise Specified	116 (87.2)
Age category	Adult	108 (91.5)
	Pediatric	10 (8.5)
Cancer Center ^1^	MSK	70 (59.3)
	UCSF	18 (15.3)
	DFCI	10 (8.5)
	MDA	5 (4.2)
	CHOP	4 (3.4)
	PROV	4 (3.4)
	VICC	2 (1.7)
	COLU	2 (1.7)
	JHU	1 (0.8)
	VHIO	1 (0.8)
	UHN	1 (0.8)
Ethnicity	Non-Hispanic	96 (81.4)
	Unknown	11 (9.3)
	Hispanic	10 (8.5)
Race	White	85 (72.0)
	Unknown	13 (11.0)
	Asian	8 (6.8)
	Black	7 (5.9)
	Other	5 (4.2)

^1^ MSK—Memorial Sloan Kettering Cancer Center, New York, NY, USA; UCSF—University of California, San Francisco, CA, USA; DFCI—Dana-Farber Cancer Institute, Boston, MA, USA; MDA—MD Anderson Cancer Center, Houston, TX, USA; CHOP—Children’s Hospital of Philadelphia, Philadelphia, PA, USA; PROV—Providence Health and Services Cancer Institute, Portland, OR, USA; VICC—Vanderbilt-Ingram Cancer Center, Nashville, TN, USA; COLU—Columbia University Medical Center, New York, NY, USA; JHU—Johns Hopkins University, Baltimore, MD, USA; VHIO—Vall d’Hebron Institute of Oncology, Barcelona, Spain; UHN—University Health Network, Toronto, ON, Canada.

**Table 2:** SWI/SNF complex mutations in chordoma.

PBRM1	ARID1A	SETD2
N258Kfs*6	D1850Gfs*4	R400*
P212Afs*3	X1709_splice	E1756*
X271_splice	A345_A349del	T2338Hfs*31
W1417*	L2088P	T2338Hfs*31
W1417*	P194L	X1485_splice
R1276Vfs*6	P1627A	A1617V
D1055Y	A247V	D1616N
D1055Y	M50V	
R1088W	W1073L	
H770P	G1770V	

* indicates a stop codon or nonsense mutation. Duplicate mutations have been highlighted in yellow.

**Table 3:** Patient demographics for chordoma NOS, conventional type chordoma, and dedifferentiated chordoma subtypes.

Demographics (Chi-Squared Test)	Category	Not Otherwise Specified N (%)	Conventional Type N (%)	Dedifferentiated N (%)	*p*-Value
Age category	Pediatric	8 (6.9)	0 (0.0)	0 (0.0)	*p* = 0.028
	Adult	108 (93.1)	14 (100.0)	3 (100.0)	
Sex	Male	64 (55.2)	5 (35.7)	3 (100.0)	*p* = 0.145
	Female	52 (44.8)	9 (64.3)	0 (0.0)	
Ethnicity	Non-Hispanic	97 (83.6)	9 (64.3)	2 (66.7)	*p* = 0.120
	Hispanic	9 (7.8)	2 (14.3)	1 (33.3)	
	Unknown	9 (7.8)	3 (21.4)	0 (0.0)	
Race	White	83 (71.6)	10 (71.4)	2 (66.7)	*p* = 0.755
	Black	6 (5.2)	2 (14.3)	0 (0.0)	
	Asian	8 (6.9)	0 (0.0)	0 (0.0)	
	Other	6 (5.2)	1 (7.1)	0 (0.0)	
	Unknown	13 (11.2)	1 (7.1)	1 (33.3)	
Sample Type	Primary	58 (50.0)	7 (50.0)	2 (66.7)	*p* = 0.678
	Metastasis	47 (40.5)	6 (42.9)	1 (33.3)	
	Other/Unknown	11 (9.5)	1 (7.0)	0 (0.0)	

**Table 4:** Patient demographics of adults and pediatric patients.

Demographics (Chi-Squared Test)	Category	Adult, N (%)	Pediatric, N (%)	*p* Value
Sex	Male	66 (54.5)	6 (50.0)	*p* = 0.503
	Female	55 (45.5)	6 (50.0)	
Ethnicity	Non-Hispanic	99 (81.8)	9 (75.0)	*p* = 0.369
	Hispanic	11 (9.1)	2 (16.7)	
	Unknown	10 (8.3)	1 (8.3)	
Race	White	91 (75.2)	4 (33.3)	*p* = 0.562
	Black	7 (5.8)	1 (8.3)	
	Asian	8 (6.6)	0 (0.0)	
	Other	7 (5.8)	0 (0.0)	
	Unknown	8 (6.6)	7 (58.3)	
Sample Type	Primary	61 (50.4)	6 (50.0)	*p* = 0.0017
	Metastasis	51 (42.1)	3 (25.0)	
	Other/Unknown	45 (37.2)	3 (25.0)	

**Table 5:** Most frequently mutated genes in primary vs. metastatic chordoma.

Sample Type	Gene	Mutations, N (%)	*p* Value
Primary	*ARID1A*	5 (7.5)	*p* = 1.00
	*PIK3CA*	5 (7.5)	*p* = 0.137
	*TP53*	4 (6.0)	*p* = 1.00
	*PBRM1*	4 (6.0)	*p* = 0.759
Metastatic	*SETD2*	6 (11.1)	*p* = 0.0411 (enriched)
	*BRCA2*	6 (11.1)	*p* = 0.0138 (enriched)
	*PBRM1*	5 (9.3)	*p* = 0.759
	*ARID1A*	5 (9.3)	*p* = 1.00

## Data Availability

The data presented in this study are available from the AACR GENIE Database at https://genie.cbioportal.org/ (accessed on 21 January 2025).

## Abbreviations

American Association for Cancer Research (AACR), Genomics Evidence Neoplasia Information Exchange (GENIE), Polybromo-1 (*PBRM1*), AT-rich interaction domain 1A (*ARID1A*), Telomerase reverse transcriptase (*TERT*), Tumor protein p53 (*TP53*), Switch/Sucrose Non-Fermentable complex (SWI/SNF complex), Breast cancer type 2 susceptibility protein (*BRCA2*), Lysine (K)-specific methyltransferase 2D (*KMT2D*), Cyclin-dependent kinase inhibitor 2A/B (*CDKN2A/B*), Phosphoinositide 3-kinase/Protein Kinase B/Mammalian Target of Rapamycin (PI3K/AKT/mTOR), Notch receptor 2 (*NOTCH2*), Coactivator-associated arginine methyltransferase 1 (*CARM1*), Parkin RBR E3 ubiquitin protein ligase (*PRKN*), Methylthioadenosine phosphorylase (*MTAP*), Enhancer of Zeste Homolog 2 (*EZH2*), Mitogen-activated protein kinase (MAPK), and Phosphoinositide-3-kinase, catalytic subunit alpha (*PIK3CA*).
